# Utilization of Left Ventricular Assist Device for Congestive Heart Failure: Inputs on Demographic and Hospital Characterization From Nationwide Inpatient Sample

**DOI:** 10.7759/cureus.16094

**Published:** 2021-07-01

**Authors:** Karnav Modi, Amanpreet K Pannu, Ronak J Modi, Suchi D Shah, Renu Bhandari, Kristal N Pereira, Khadija T Kubra, Maharshi R Raval, Temitope Ajibawo

**Affiliations:** 1 Internal Medicine, Byramjee Jeejeebhoy Medical College, Ahmedabad, IND; 2 Medicine, Sri Guru Ram Das University of Health Sciences, Amritsar, IND; 3 Cardiology, Bankers Heart Institute, Vadodara, IND; 4 Internal Medicine, Ahmedabad Municipal Corporation's Medical Education Trust Medical College, Ahmedabad, IND; 5 Medicine, Manipal College of Medical Sciences, Kaski, NPL; 6 Internal Medicine, Terna Medical College, Mumbai, IND; 7 Internal Medicine, Bangladesh Medical College, Dhaka, USA; 8 Internal Medicine, Brookdale University Hospital Medical Center, New York City, USA

**Keywords:** management of heart failure, lvad, left ventricular assist device, demographics, utilization management, in-hospital outcome

## Abstract

Objectives

The first goal of the study is to provide a descriptive overview of the utilization of left ventricular assist device (LVAD) for the treatment of congestive heart failure (CHF) and determine the rates of LVAD use stratified by patients’ demographic and hospitals’ characteristics in the United States. Next, is to measure the hospitalization outcomes of length of stay (LOS) and cost in inpatients managed with LVAD.

Methods

We conducted a cross-sectional study using the nationwide inpatient sample and included 184,115 patients (age ≥65 years) with a primary discharge diagnosis of hypertensive and non-hypertensive CHF and was further classified by inpatients who were managed with LVAD. We compared the distributions of demographic and hospital characteristics in CHF inpatients with versus without LVAD by performing Pearson’s chi-square test for categorical variables, and independent sample t-test for continuous variables.

Results

The inpatient utilization of LVAD was 0.93% (1690 out of 184,115) in CHF patients. The LVAD cohort were younger compared to non-LVAD group (mean age, 69.9 years vs. 79.4 years). The utilization rate of LVAD was also almost four times higher in males (1.50%) compared to females (0.36%). Although whites (78.5%) accounted for majority of LVAD recipients, the rate of LVAD utilization was highest in blacks (1.04%) and lowest in Hispanics (0.58%) with whites having utilization rate of 0.89%. Medicare was the dominant primary payer to cover the LVAD inpatients (91.1%), though the rate of LVAD utilization is highest in private (2.22%) and lowest in those covered by public insurance (medicaid/medicare). CHF patients in public hospitals (1.79%) were more than twice more likely to receive LVAD than in private hospitals (0.83%) due to higher utilization rate. LVAD utilization rate was approximately 55 times higher in teaching hospitals (1.67%) compared to non-teaching hospitals (0.03%), and 20 times higher in large bed hospitals (1.41%) compared to small bed-size hospitals (0.07%). CHF patients that received LVAD had a significantly longer LOS (34.6 days vs 9.8 days) and higher inpatient treatment costs ($802,118 vs. $86,302) compared to non-LVAD group.

Conclusion

The inpatient utilization of LVAD was in CHF patients is higher in males, blacks and private health insurance beneficiaries. In terms of hospital characteristics, the utilization of LVAD for CHF management was higher in large bed sized, and public type and teaching hospitals compared to their counterparts. This data will allow us to devise strategies to improve LVAD utilization and increase its outreach for heart failure patients, especially those on the transplant waiting list. Despite its effectiveness, aggressive usage of LVAD is restricted due to cost-effectiveness and lack of technical confidence among medical professional due to complications.

## Introduction

American Heart Association (AHA) estimated that there were 6.2 million people with congestive heart failure (CHF) in the United States (US) between 2013 and 2016 [1]. There are an estimated 23 million people with HF worldwide [2]. The Framingham heart study found a prevalence of CHF in men of eight per 1,000 at age 50 to 59 years, increasing to 66 per 1,000 at ages 80 to 89 years; similar values (eight and 79 per 1,000) were noted in women [3]. Improved treatment of hypertension, valvular, and coronary disease is allowing patients to survive an early death only to later develop CHF. The prevalence of CHF in the US is projected to rise over the next four decades, with an estimated 772,000 new CHF cases projected in 2040 [4].

Left ventricular assist device (LVAD) is considered for two groups of patients, one pertains to patients with acute cardiogenic shock and the other includes patient with inotrope-dependent CHF. For the patients with acute cardiogenic shock, mechanical circulatory support (MCS) is indicated if the patient has ventricular function which is unrecoverable without MCS. Ejection fraction <25% and reduced functional capacity are strong indications for the need for LVAD [5]. In addition, one-year mortality of the patients with CHF should be calculated with prognostic models and high-risk patients should be considered for LVAD [6].

The concept of MCS developed concomitantly with the field of heart transplantation (HT), as LVADs were originally conceived as temporary bridge-to-transplant (BTT) platforms. Initially, BTT was an effective strategy to rescue patients whose severity of CHF precluded survival on medical therapy alone until a donor organ became available. However, it was recognized early on that utilizing LVAD solely as BTT was inadequate in addressing the growing prevalence of end-stage CHF. With the annual volume of HT in the US stagnating around 3000, implanting LVAD as BTT without addressing this bottleneck only increased the size of the BTT population vying for HT. The proportion of those who received HT as BTT increased from 19.1% in 2000 to 41.0% in 2012 [7]. Patients treated with LVAD have better survival and improved functional quality of life when compared to those treated medically [8,9].

There is limited data on the utilization rate of LVAD in the United States, and so we conducted a cross-national study. Our first goal is to provide a descriptive overview of the utilization of LVAD for the treatment of CHF and determine the rates of LVAD use stratified by patients’ demographic and hospitals’ characteristics in the United States. Our second goal is to measure the hospitalization outcomes including length of stay (LOS) and cost in inpatients managed with LVAD.

## Materials and methods

Study sample

We conducted a cross-sectional study using the nationwide inpatient sample (NIS). The NIS is the inpatient data obtained from 4,411 hospitals across 44 states in the US. The NIS is a de-identified data with the protection of patient’s health information, and so we were not required to take institution review board permission for our study [10].

We included 184,115 elderly patients (age ≥65 years) with a primary discharge diagnosis of hypertensive and non-hypertensive CHF and having major severity of illness as per the variable in the NIS. The sample was further grouped by inpatients who were managed with LVAD as the primary procedure.

Variables

Demographic variables included were age, sex, race, primary payer, and region. Hospital variables drawn from the American Hospital Association (AHA) annual survey of hospitals included hospital ownership (public or private), bed size (small, medium, or large), location (urban or rural), and teaching status (teaching or non-teaching). We included the LOS and total cost during hospitalization for the treatment of CHF as provided in the NIS [11].

Statistical analysis

We compared the distributions of demographic and hospital characteristics in CHF inpatients with versus without LVAD by performing descriptive statistics and Pearson’s chi-square test. Next, we measured the differences in continuous variables i.e., age, LOS and total cost in inpatients with and without LVAD using the independent sample t-test. All analyses were conducted using Statistical Package for the Social Sciences (SPSS) version 26.0 (IBM Corp., Armonk, NY) and statistical significance was set at a two-sided P value <0.05.

## Results

The inpatient utilization of LVAD was 0.93% (1,690 out of 184,115) in CHF patients. The LVAD cohort were younger compared to non-LVAD group (mean age, 69.9 years vs. 79.4 years). Males accounted for majority of LVAD recipients (79.4%) and the utilization rate of LVAD was also almost four times higher in males (1.50%) compared to females (0.36%). Although whites (78.5%) accounted for majority of LVAD recipients followed by blacks (13.7%), and hispanic (6.4%), the rate of utilization was highest in blacks (1.04%) and lowest in hispanics (0.58%) with whites having utilization rate of 0.89%.

Medicare was the dominant primary payer to cover the LVAD inpatients (91.1%) followed by private (six percent), self-pay (1.5%) and Medicaid 1.3%. But surprisingly, the rate of utilization is highest in private (2.22%) and lowest in Medicaid (0.43%) with medicare and self-pay having utilization rates of 0.83% and 0.88% respectively. Utilization of LVAD varied widely by the US geographic region; the south (40.5%) had the highest LVAD recipients, followed by the midwest (28.7%), the northeast (20.4%) and the west (10.4%). The west region of the US also had the lowest utilization rate of LVAD (0.57%) and rest of other regions have similar utilization rates (midwest 1.15%, south 0.94%, northeast 0.91%).

Although private hospitals accounted for majority of LVAD recipients (83.1%), CHF patients in public hospitals (1.79%) were more than twice more likely to receive LVAD than in private hospitals (0.83%) due to higher utilization rate. Similarly, larger bed-size (89.1%) and teaching hospitals (98.2%) accounted for most of LVAD recipients and LVAD utilization rate was approximately 55 times higher in teaching hospitals (1.67%) compared to non-teaching hospitals (0.03%). Compared to small bed-size hospitals (0.07%), utilization rate was around five times higher in medium bed-size (0.33%) and 20 times higher in large bed hospitals (1.41%) as shown in Table 1.

**Table 1:** Demographic and hospital characteristics of inpatients by utilization of left ventricular assist device. LVAD: left ventricular assist device.

Variable	LVAD (no)	LVAD (yes)	Rate of LVAD (%)	P-value
N	182425	1690	0.93	-
Mean age	79.4	69.9	-	<0.001
Sex, in %				
Male	48.6	79.9	1.50	<0.001
Female	51.4	20.1	0.36	
Race, in %				
White	77.1	78.5	0.89	<0.001
Black	11.4	13.7	1.04	
Hispanic	6.4	4.2	0.58	
Other	5.1	3.6	0.61	
Primary payer, in %				
Medicare	91.1	83.0	0.83	<0.001
Medicaid	1.3	0.6	0.43	
Private	6.0	14.9	2.22	
Self-pay/uninsured	1.5	1.5	0.88	
Region				
Northeast	20.7	20.4	0.91	<0.001
Midwest	22.8	28.7	1.15	
South	39.7	40.5	0.94	
West	16.8	10.4	0.57	
Ownership of hospital				
Public	8.5	16.9	1.79	<0.001
Private	91.5	83.1	0.83	
Bed size of hospital				
Small	14.9	1.2	0.07	<0.001
Medium	27.3	9.8	0.33	
Large	57.8	89.1	1.41	
Hospital teaching status				
Non-teaching	46.4	1.8	0.03	<0.001
Teaching	53.6	98.2	1.67	

CHF patients that received LVAD had a significantly longer LOS (34.6 days vs 9.8 days) and higher inpatient treatment costs compared to non-LVAD group. Also, the mean cost for LVAD inpatients was $802,118 which was around nine times higher than that of non-LVAD group ($86,302) as shown in Table 2.

**Table 2:** Hospitalization outcomes of inpatients by utilization of left ventricular assist device. LVAD: left ventricular assist device.

Outcome	LVAD (no)	LVAD (yes)	P-value
Mean length of stay, days	9.8	34.6	<0.001
Mean cost, $	86302.5	802118.2	<0.001

## Discussion

Age is an independent predictor of outcomes for post-LVAD survival, with the older population more likely to have complications as well as increased morbidity and mortality [12]. This is the reason LVAD implantation is preferred in the lower age group, as noted in our study, with higher age group people managed medically or with orthotopic heart transplantation (OHT). Our study found that males were more likely to get LVAD implantation compared to females by a ratio of 4:1. This may partly be due to the conventional understanding that large, pulsatile flow LVADs do not fit the small-sized body of women and their small chamber size [13].

There are conflicting reports regarding in-hospital mortality and outcomes differences after LVAD implantation based on sex. The eighth annual interagency registry for mechanically assisted circulatory support (INTERMACS) report showed the female sex to be a risk factor for early mortality [14]. However, other studies did not show any gender differences in in-hospital mortality or early mortality with continuous-flow LVAD [15,16]. Even with the advances in the size and dynamics of LVAD, women continue to have an underrepresentation in getting LVADs. They are more likely to be on a waitlist longer for a heart transplant and suffer clinical deterioration during this period [17].

Although the percentage of blacks amongst people undergoing LVAD implantation is much less than whites, the rate of LVAD utilization in blacks is more than whites, hispanics, and other races. This observation is similar to findings by previous studies which showed increased LVAD implantation rates among blacks in comparison to other race/ethnicities [18,19]. The reason may be due to insurance coverage increases amongst blacks in recent years [20]. Private insurance may play a significant role, with the rate of LVAD utilization being highest amongst patients holding private insurance based on our results. Patients with medicaid were less likely to get LVAD according to our study and this finding is similar to previous studies [21,22].

Private ownership of the hospital had higher LVAD implantation by a ratio of more than 4:1 as supported by a previous study [23]. However, the reason behind the increased rate of use of LVAD in public hospitals is not entirely clear. Most public hospitals being larger sized, teaching hospitals with better logistics to deal with early complications and readmissions may be one of the reasons behind the increased rate of LVAD’s use in public hospitals [24].

History of coronary artery bypass graft (CABG) or valve surgery, diabetes, ascites, INTERMACS profiles one and two, and low albumin, high blood urea nitrogen (BUN), and high right atrial pressure are associated with high chances of prolonged hospital stay [25]. A study by Sheribati et al. assessed the mean cost of LVAD implantation to be $175,000. While considering the costs of hospital re-admissions and outpatient clinic visits, the incremental cost-effectiveness ratio (ICER) was found to be $209,400 per quality-adjusted life-year (QALY) gained and $597,400 per life-year gained [26]. These could be possible reasons for higher LOS and cost of hospitalization for the CHF patients receiving LVAD treatment in our study.

Our study results should be considered with some limitations. Firstly, the NIS is an administrative database in which variables were identified by the diagnostic coding that is subject to coding inaccuracies and underreporting of comorbidities. NIS lacks detailed patient-level information such as INTERMACS classification, and the pattern of anticoagulation use. This is a cross-sectional study and so a causal relationship cannot be established for factors influencing the utilization of LVAD and associated hospital outcomes. Yet, the NIS offers an incomparable population-based perception of utilization of procedures in a nationally representative inpatient sample. The information is coded independently by individual practitioners and is less likely to have reporting bias.

## Conclusions

In this new era of technology, MCS devices such as the LVAD will make an immense difference for patients with refractory CHF, and as a durable alternative to a heart transplant. The inpatient utilization of LVAD was in CHF patients is higher in males, blacks and private health insurance beneficiaries. In terms of hospital characteristics, the utilization of LVAD for CHF management was higher in large bed-sized, and public type and teaching hospitals compared to their counterparts. This data will allow us to devise strategies to improve LVAD utilization and increase its outreach for heart failure patients, especially those on the transplant waiting list. Despite its effectiveness, aggressive usage of LVAD is restricted due to cost-effectiveness and lack of technical confidence among medical professionals due to complications. Hence, the indications and usefulness of widespread use of LVAD should be studied with clinical trials enrolling a large number of patients so that more eligible patients can benefit from this therapy and improve their prognosis.
